# Simultaneous Robot‐Assisted Radical Prostatectomy and Repair of Inguinal Bladder Hernia

**DOI:** 10.1002/iju5.70008

**Published:** 2025-02-24

**Authors:** Naoki Imasato, Shugo Yajima, Ryo Andy Ogasawara, Minoru Inoue, Kohei Hirose, Madoka Kataoka, Yasukazu Nakanishi, Hitoshi Masuda

**Affiliations:** ^1^ National Cancer Center Hospital East Chiba Japan

**Keywords:** bladder hernia, hernia repair, inguinal hernia, prostate cancer, prostatectomy

## Abstract

**Introduction:**

We report the first case of simultaneous robot‐assisted radical prostatectomy (RARP) and inguinal bladder hernia repair in a patient with prostate cancer, highlighting the feasibility and technical considerations of a combined robotic approach.

**Case Presentation:**

A 70‐year‐old male underwent simultaneous RARP and inguinal hernia repair using a robotic approach. A 3D Max mesh (Bard Inc., New Providence, NJ) was placed to reinforce the hernia repair. Postoperatively, the patient has shown no recurrence of the hernia or signs of infection during follow‐up.

**Conclusion:**

Simultaneous RARP and bladder hernia repair using a robotic approach was successfully performed without complications. This combined procedure may be a safe and effective surgical option for similar cases requiring dual interventions.

**Summary**

- First reported case of robot‐assisted radical prostatectomy (RARP) combined with bladder hernia repair.
- The robotic approach provides enhanced visualization and precision.
- Simultaneous procedure reduces patient burden and overall costs.
- Successful application of RARP and bladder hernia repair in a patient with coexisting bladder hernia and prostate cancer.

## 1 Introduction

Bladder hernias are uncommon clinical conditions, accounting for only 1%–4% of all inguinal hernias [1, 2, 3]. Their coexistence with urological malignancies, including prostate cancer, has been reported in the literature and is not an exceptional occurrence [4]. These hernias predominantly affect males over the age of 50, and often present with symptoms such as a groin bulge, voiding difficulties, or other lower urinary tract symptoms (LUTS). The pathophysiology of bladder hernias involves bladder outlet obstruction, obesity, and reduced bladder tone and weakened pelvic muscles common in older adults [3]. The standard treatment for inguinal bladder hernias is surgical repair with mesh placement to prevent recurrence [5].

Cases of bladder hernias concurrent with genitourinary malignancies, such as bladder and prostate cancers, have been reported [6, 7]. However, there are no reports of patients undergoing simultaneous robot‐assisted radical prostatectomy (RARP) with bladder hernia repair. This report presents the first case of a patient with PCa and a bladder hernia who successfully underwent simultaneous RARP and bladder hernia repair using a robot‐assisted approach.

## 2 Case Presentation

**FIGURE 1:**
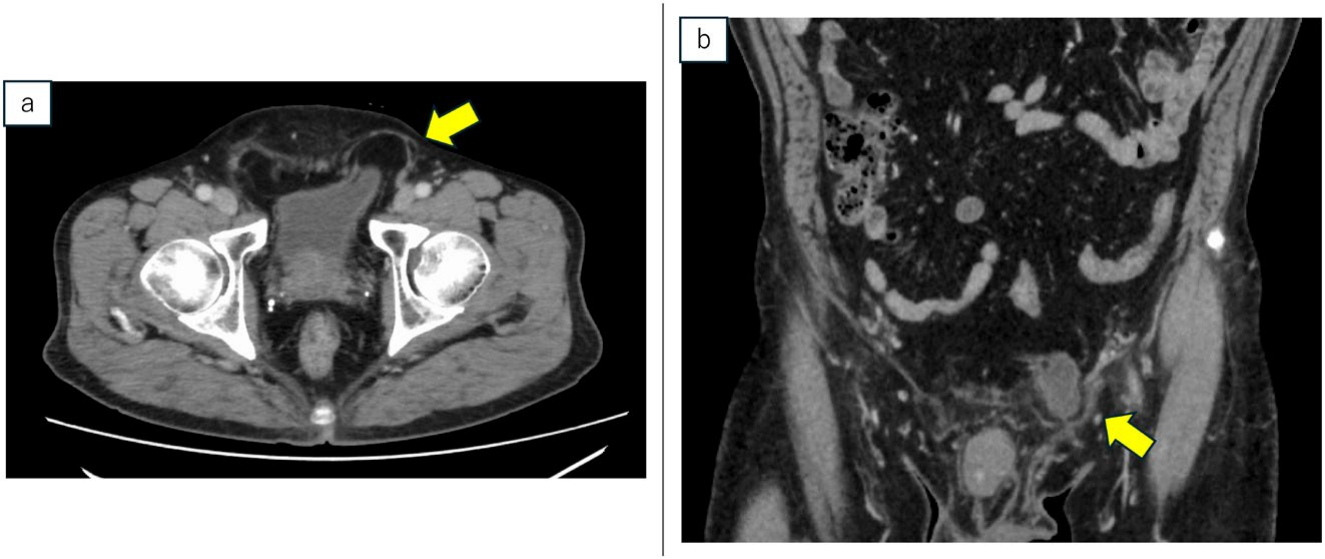
(a) Axial and (b) coronal computed tomography scan revealed left direct bladder hernia with a part of the bladder protrusion.

A 70‐year‐old Japanese male with a medical history of hypertension, appendicitis, and childhood inguinal hernia repair (side unknown) presented with left inguinal discomfort. He was referred for further evaluation following elevated prostate‐specific antigen (PSA) level of 5.24 ng/mL. Magnetic resonance imaging (MRI) revealed no malignancy. Preoperative imaging measured the prostate volume at 40 cm^3^. A Transperineal prostate biopsy confirmed prostatic adenocarcinoma with a Gleason score of 3 + 4 in the left lobe of the prostate. Computed tomography (CT) revealed a part of the bladder protruding as a direct hernia, establishing the diagnosis of a bladder hernia (Figure 1). The patient had no history of LUTS or urinary retention. Bone scans and CT imaging indicated no evidence of metastasis. Based on these findings, he was referred to our institution for surgical management. A simultaneous RARP and inguinal bladder hernia repair were planned. To minimize the risk of postoperative infection, a preoperative urine culture was performed, which was negative.

## 3 Surgical Procedure

**FIGURE 2:**
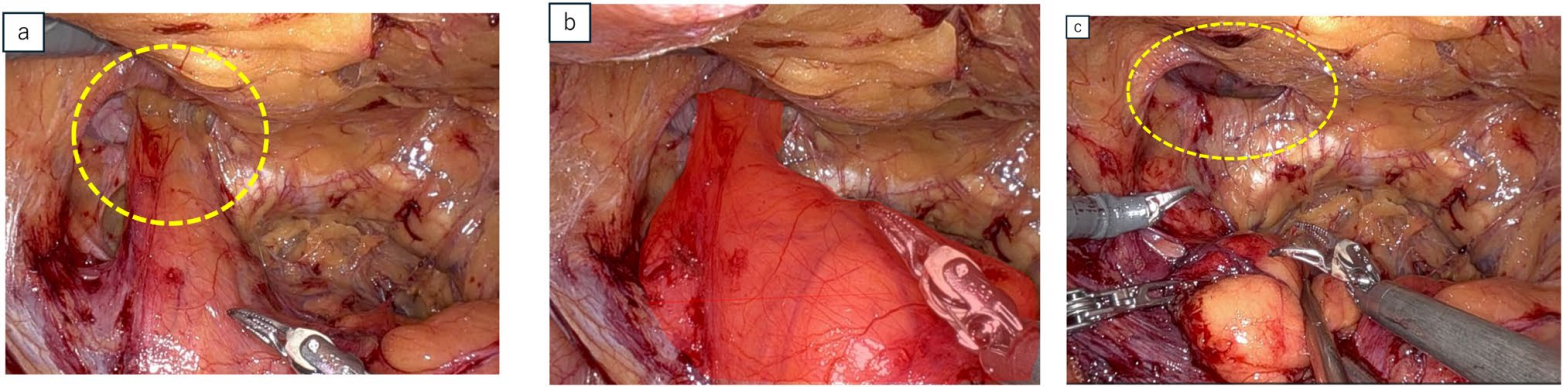
(a) Intraoperative view revealed the direct inguinal hernia containing the bladder (broken yellow circle). (b) The bladder identified after saline instillation to confirm its contours (outlined in the red field). (c) Broken yellow circle indicates the hernial orifice.

**FIGURE 3:**
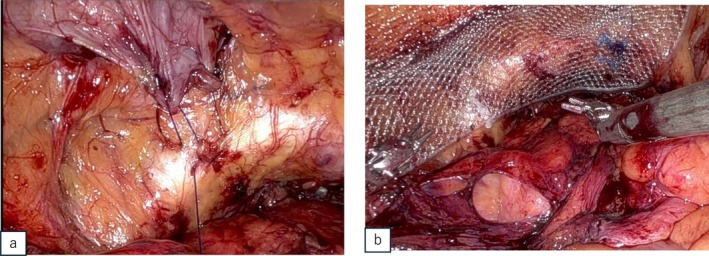
(a) Fixation of the transversalis fascia to Cooper's ligament using barbed suture. (b) 3D Max mesh was fixed over the hernial orifice using two stitches.

The procedure was performed by a highly skilled surgeon the Da Vinci Xi surgical system (Intuitive Surgical Inc., Sunnyvale, CA, USA) with a six‐port intraperitoneal approach. A horizontal incision was made in the peritoneum, followed by lateral dissection. While accessing the Retzius' cavity, a left direct inguinal hernia with a protruding bladder was identified, confirming that a portion of the bladder had extended into the hernial orifice (Figure 2a). Saline injection into the bladder reaffirmed that the protrusion originated from the bladder (Figure 2b). The protruding bladder walls were carefully repositioned to their positions. The hernia sac was isolated from the protruding bladder, exposing the hernial orifice (Figure 2c). Prostatectomy and vesicourethral anastomosis were subsequently performed. The transversalis fascia was sutured to Cooper's ligament using a 3–0 barbed suture (Figure 3a). A 3D Max mesh (Bard Inc., New Providence, NJ) was placed over the hernial orifice, secured with two stitches (Figure 3b), and the peritoneal flap was repaired with continuous suture.

The total operative time was 159 min, with a console time of 128 min, including 19 min for hernia repair. Estimated blood loss was 314 mL.

## 4 Postoperative Details

Urethrocystography on postoperative day 6 indicated no leakage at the vesicourethral anastomosis site. The patient was discharged 9 days postoperatively without complications (Clavien‐Dindo grade 0).

**FIGURE 4:**
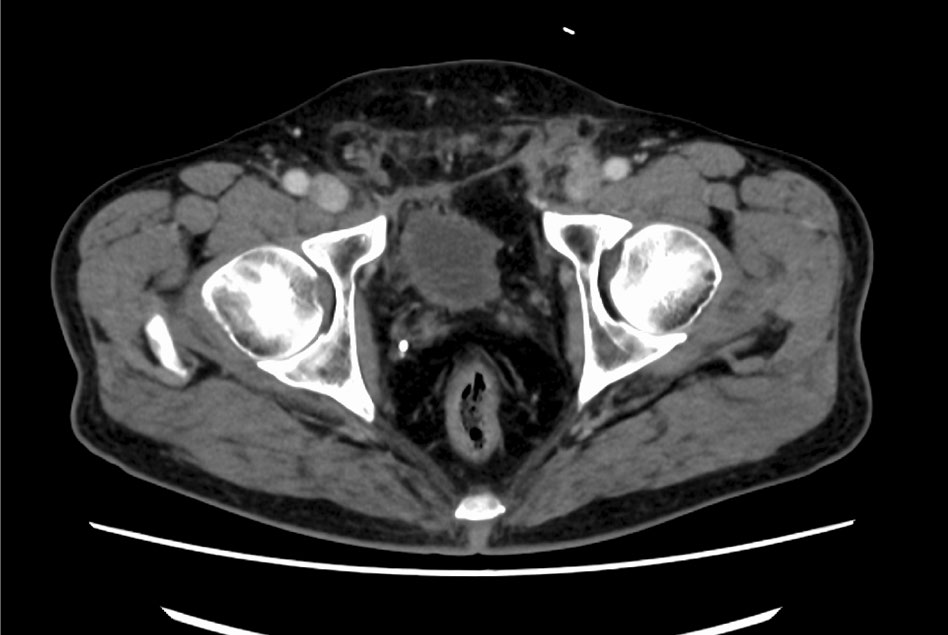
Follow‐up computed tomography scan at 1 month showed that the bladder hernia had disappeared.

Additionally, the patient's inguinal discomfort was resolved postoperatively. Pathological examination confirmed adenocarcinoma with a Gleason score of 3 + 3 in both lobes, with negative surgical margins. Follow‐up CT at 1 month demonstrated no recurrence of the hernia (Figure 4), and the patient remained symptom‐free. Over a 3‐month period, the patient indicated no signs of mesh infection or PSA recurrence and reported being pad‐free.

## 5 Discussion

In this case, a 70‐year‐old male with both PCa and bladder hernia successfully underwent simultaneous RARP and hernia repair. In standard practice, bladder hernias and PCa are managed independently. Traditional methods for managing bladder hernias involve open or laparoscopic hernia repair [8].

Robot‐assisted techniques have become increasingly common in inguinal hernia repair, with several studies demonstrating that combined RARP and inguinal hernia repair is safe and feasible [9, 10, 11]. Our previous findings have reported no increase in postoperative complications with this approach [12]. However, no prior reports exist on the concurrent use of robot‐assisted techniques for managing both PCa and bladder hernias.

In bladder hernia cases, robotic approach enables precise observation of the protruding bladder. The robotic simultaneous approach offers several advantages, including enhanced visualization of the compressed bladder, smoother surgical execution, and greater precision compared to a two‐step approach. Furthermore, performing both procedures in a single operation reduces overall costs and patient burden. This combined approach avoids postoperative changes in the abdominal wall or inguinal region that might occur following prostatectomy.

This procedure posed several challenges, including the need for precise dissection to avoid bladder injury while ensuring adequate prostate resection. To facilitate safe dissection, saline was instilled into the bladder to delineate its contours. This technique was particularly useful for identifying and safely mobilizing the portion of the bladder. Fortunately, the bladder and the hernia sac were straightforward to separate, allowing for efficient dissection and minimizing the risk of injury. Given the potential risk of bladder injury, careful preoperative evaluation and intraoperative strategies, such as saline instillation, are essential to ensure safe dissection and optimal surgical outcomes.

During the hernia repair, we fixed the transversalis fascia to Cooper's ligament and placed a mesh. Fixation of the transversalis fascia to Cooper's ligament reduces dead space, potentially preventing postoperative complications such as seroma [13, 14].

Concerns exist regarding mesh infection if vesicourethral anastomotic leakage occurs during simultaneous RARP and hernia repair. However, previous studies [15, 16] have reported no cases of mesh infection requiring removal, even in large cohorts, despite the theoretical risk. Routine cystography before Foley catheter removal plays a crucial role in detecting leaks and minimizing mesh exposure to urine. Additionally, in this case, a negative preoperative urine culture helped mitigate infection risk.

Hernia repair also resolved the patient's groin discomfort. Although this patient did not present LUTS preoperatively, bladder hernia repair may potentially relieve LUTS in symptomatic patients. This report indicates that combining RARP and bladder hernia repair may be a safe and effective approach. However, further controlled, prospective studies are required to confirm the safety and efficacy of this combined approach. Additionally, our follow‐up period was short, and long‐term data are necessary to assess the recurrence of hernia and validate the durability of the outcomes. A limitation of this case is the rarity of bladder hernia coexisting with PCa, making it challenging to generalize the outcomes.

## 6 Conclusion

Our experience demonstrates the safe and effective management of simultaneous RARP and bladder hernia repair in patients with dual diagnoses, emphasizing the potential benefits of robot‐assisted surgery. Although our findings suggest that this combined approach is feasible with minimal morbidity, additional studies are required to validate long‐term outcomes and assess its broader applicability.

## Consent

Written informed consent was obtained from the patient.

## Conflicts of Interest

The authors declare no conflicts of interest.
